# Molecularly Imprinted Electrochemical Sensor Based on MWCNTs/GQDs for the Detection of Sulfamethazine in Aquaculture Seawater

**DOI:** 10.3390/bios15030184

**Published:** 2025-03-13

**Authors:** Jianlei Chen, Tianruo Zhang, Yong Xu, Hao Li, Hongwu Cui, Xinguo Zhao, Yun Zhou, Keming Qu, Zhengguo Cui

**Affiliations:** 1 State Key Laboratory of Mariculture Biobreeding and Sustainable Goods, Yellow Sea Fisheries Research Institute, Chinese Academy of Fishery Sciences, Qingdao 266071, China; chenjl@ysfri.ac.cn (J.C.);; 2 Laboratory for Marine Fisheries Science and Food Production Processes, Qingdao Marine Science and Technology Center, Qingdao 266237, China; 3 College of Fisheries and Life Science, Dalian Ocean University, Dalian 116023, China

**Keywords:** aquaculture seawater, sulfadimidine, electrochemical sensor, molecular imprinting

## Abstract

In this work, a novel molecularly imprinted electrochemical sensor was proposed based on molecular imprinting technology for the detection of sulfamethazine. A glassy carbon electrode was modified with a composite material of carbon nanotubes and graphene quantum dots to effectively improve sensitivity. The molecularly imprinted electrochemical sensor was then prepared by electropolymerization using sulfamethazine as the template and o-phenylenediamine as the functional monomer on the modified electrode. Under optimal measurement conditions, electrochemical tests of different sulfamethazine concentrations (0.5 μM–200 μM) showed excellent linearity and a detection limit of 0.068 μM. In addition, the sensor demonstrated satisfactory selectivity, stability, and reusability. Furthermore, the sensor was applied to the spiked analysis of sulfamethazine in grouper aquaculture water, achieving recovery rates between 95.4% and 104.8%, with a relative standard deviation (RSD) of less than 4.14%. These results indicated that the developed method was effective for the analysis of sulfamethazine in aquaculture seawater, providing a new approach for the detection of antibiotic residues in seawater samples.

## 1. Introduction

Antibiotics are essential compounds widely used in both human and veterinary medicine to prevent and treat bacterial infections [1,2]. Beyond medical applications, antibiotics play a significant role in animal husbandry and aquaculture, where they help control bacterial diseases and promote growth. However, the widespread and often unregulated use of antibiotics has raised serious environmental concerns, particularly in aquatic ecosystems [3,4]. In aquaculture, 20% to 30% of antibiotics administered to fish and other aquatic organisms are absorbed, while the remaining portion is excreted into the surrounding water [5,6]. The extensive use of antibiotics has resulted in their persistent detection across aquatic environments [7,8,9], creating multi-tiered ecological risks. Untreated antibiotic discharges from aquaculture operations directly accumulate in marine ecosystems [10], exacerbating contamination levels that threaten environmental stability. More critically, these residues disrupt the microbial equilibrium [11], induce the proliferation of antibiotic-resistant bacterial strains [12,13], and exhibit toxicological impacts on non-target marine organisms [14,15,16].

Sulfamethazine (SMZ) is a synthetic sulfonamide antibiotic known for its broad-spectrum antibacterial activity, chemical stability, ease of administration, and low cost, making it widely used in aquaculture. To safeguard human health and safety, specific sulfonamide antibiotics such as sulfathiazole and sulphaguanidine have been prohibited for application in fish disease prevention and as feed additives in aquatic farming. Residual SMZ in water is particularly concerning due to its persistence and potential to induce antibiotic resistance. To safeguard public health and protect ecosystems, the Codex Alimentarius Commission (CAC) and regulatory agencies in various countries have established maximum residue limits (MRLs) for sulfonamides in food, including SMZ [17,18]. The established MRL for SMZ is typically 0.1 mg/kg in food, underscoring the need for accurate and reliable detection methods in both food and environmental samples [19,20].

Traditional analytical techniques for detecting antibiotic residues, such as high-performance liquid chromatography (HPLC) and liquid chromatography–mass spectrometry (LC-MS), are well established and highly sensitive [21,22,23,24]. However, these methods tend to be labor intensive, requiring extensive sample pretreatment and expensive instrumentation, which limits their practicality for routine monitoring in aquaculture environments [25,26]. Additionally, the complexity of the seawater matrix, with its high salinity and the presence of various compounds, poses additional challenges for the accurate detection of antibiotic residues [27].

In response to these limitations, electrochemical sensors have emerged as a promising alternative for detecting antibiotics in complex matrices like seawater [25]. Electrochemical sensors offer several advantages, including high sensitivity, fast response times, and the ability to be miniaturized for on-site and real-time monitoring [28,29]. Among the various electrochemical sensing technologies, molecular imprinting technology has attracted attention for its ability to create highly selective recognition sites for target analytes [30,31]. Molecularly imprinted polymers (MIPs) mimic biological recognition processes by forming specific binding sites tailored to the shape, size, and functional groups of target molecules [32,33]. This capability enables the selective identification of specific compounds, even amidst structurally similar substances or other potential interferences.

After the modification of MIPs on the electrode surface, electron transfer and diffusion are somewhat hindered, reducing the efficiency of molecularly imprinted electrochemical sensors. To enhance the performance of these sensors, the addition of nanomaterials is one of the most effective approaches. Nanomaterials such as multi-walled carbon nanotubes (MWCNTs) and graphene quantum dots (GQDs) have been incorporated into sensor designs [34,35].

Carbon nanotubes (CNTs) are derivatives of carbon fibers and fullerenes, consisting of sp^2^ hybridized orbitals between carbon atoms. Based on the number of layers, CNTs are primarily classified into two categories: single-walled carbon nanotubes (SWCNTs) and multi-walled carbon nanotubes (MWCNTs). MWCNTs are composed of cylinders concentrically rolled up by two or more graphene sheets and exhibit better chemical stability, strong adsorption properties, high mechanical strength, and excellent electrical conductivity [36]. While the performance of SWCNTs is similar to that of MWCNTs, MWCNTs are more suitable for electrochemical applications, whereas SWCNTs are more suited for optical applications [37].

GQDs are an emerging class of carbon nanoparticles, consisting of fewer than five layers of 1.5–100 nm graphene sheets with predominantly circular and elliptical shapes. Like graphene, GQDs exhibit the characteristics of both graphene and carbon dots, composed of carbon (C), hydrogen (H), and oxygen (O), with surface groups including carbonyl, carboxyl, hydroxyl, and epoxy. Their carbon atoms are linked by sp^2^ hybridization [38,39]. GQDs have broad application prospects in bioimaging, photoluminescence, catalysis, and fluorescent sensors due to their high fluorescence activity, good water solubility, high specific surface area, excellent electrical conductivity, high mobility, and good biocompatibility. In addition, GQDs are considered to be good electron donors and acceptors, making them suitable materials for electrode fabrication [40].

MWCNTs and GQDs possess exceptional electrical conductivity, large surface areas, and high chemical stability, which significantly improve the sensitivity and signal-to-noise ratio of sensors [41,42,43,44,45].

Notably, significant advances have been achieved in molecularly imprinted polymer (MIP)-based sensors. To systematically evaluate their application in sulfamethazine (SMZ) monitoring, we have compiled recent progress in MIP sensor development for SMZ detection, with key findings summarized in Table S1 (see Supplementary Materials) [28,46,47,48,49,50,51,52,53,54,55,56]. Various molecularly imprinted polymer (MIP)-based electrochemical sensors have been developed for antibiotic detection, but their application in complex environments such as seawater remains limited. The proposed MIP sensor achieves a very low detection limit which was significantly lower than those reported in earlier works using fluorescence-only sensors. However, Zhang et al. [53] successfully achieved the detection of five sulfa antibiotics in aquaculture water and fish species using a fluorescence sensor array constructed based on photonic crystal molecular imprinted polymers, with a relatively lower detection limit (0.01–0.26 nM), which represented a milestone in multi-analyte detection within aquaculture-related matrices. In addition, although numerous studies have reported MIP-based electrochemical sensors for antibiotic detection, most of these works focus primarily on tap water or food. The stability and selectivity of such sensors in high-salinity, complex aquaculture seawater remain a significant challenge. For instance, the MIP sensor developed achieved a lower detection limit in meat; however, its performance in seawater had not been validated [52]. In contrast, the proposed sensor was specifically optimized for aquaculture seawater conditions, ensuring stable SMZ detection even under high-salinity conditions. Building upon prior methodologies for antibiotic detection, this study established a new method for SMZ detection in aquaculture seawater—a matrix with inherent complexity due to high salinity and organic interference.

In this study, a molecularly imprinted electrochemical sensor based on multi-walled carbon nanotubes and graphene quantum dot (MWCNTs/GQD) composites were innovatively proposed for the detection of sulfamethazine in aquaculture seawater. The sensor utilized a glassy carbon electrode (GCE) modified with MWCNTs/GQD composites. A pair of molecularly imprinted membranes were then modified onto the electrode surface through electropolymerization, using sulfamethazine as the template molecule. The surface of the molecularly imprinted membranes forms highly selective recognition sites, enabling the specific detection of SMZs even in the presence of potentially interfering substances commonly found in seawater. Key parameters affecting the sensor’s performance, including the amount of MWCNTs/GQD coating, the pH of the electrolyte, and conditions for polymer deposition, were systematically optimized to enhance its detection capabilities. The analytical performance of the sensor was evaluated in terms of its linear detection range, detection limit, and selectivity. Finally, the sensor was validated by applying it to the detection of SMZ in spiked seawater samples collected from aquaculture systems.

## 2. Materials and Methods

### 2.1. Reagents and Apparatus

N,N-dimethylformamide (DMF, ≥99.5%) was purchased from Kemel Chemical Company (Tianjin, China). Sulfamethazine (SMZ, ≥99.5%), Multi-walled carbon nanotubes (MWCNTs, ≥95%, ID: 3–5 nm, OD: 8–15 nm, Length: 0.5–2 μM) and graphene quantum dots (GQDs) were all obtained from Macklin Biochemical Co., Ltd. (Shanghai, China), and the XRD of them is shown in Figure S1. o-Phenylenediamine (OPD, ≥99.5%) was purchase from Aladdin Reagent Co., Ltd. (Shanghai, China). Potassium ferricyanide (K_3_[Fe(CN)]_6_, ≥99.5%), potassium chloride (KCl, ≥99.5%), and potassium ferrocyanide (K_4_[Fe(CN)]_6_·3H_2_O, ≥99.5%) were purchased from Sinopharm Chemical Reagent Co., Ltd. (Shanghai, China). Ultrapure water (18.25 MΩ·cm^−1^) was used throughout the experiment.

All electrochemical studies were performed with a CHI660 workstation which composed of a glassy carbon electrode (GCE, 3 mm in diameter) as working electrode (CH Instruments, Shanghai, China). An ultrasonic cleaning machine was purchased from Kunshan (KQ3200V, China). Acquired amounts were weighed in the precision balance by Ohaus Instruments (Shanghai, China). The pH of the solutions was measured with a pH meter (PHSJ-6L, Shanghai, China).

### 2.2. MWCNTs/GQD Coating on the GCE

Firstly, 50 mg of MWCNTs and 50 mg of GQDs were dispersed in 50 mL of DMF. After 2 h of ultrasonic cleaning, the dispersion of the composite carbon nanomaterials was obtained. Secondly, dissolve 1.0 g of chitosan powder in 100 mL of 1% acetic acid solution and sonicate for 30 min. Finally, mix the dispersed carbon nanomaterials with chitosan solution and sonicate to homogenize for 2 h, resulting in a composite carbon nanotube/graphene quantum dot (MWCNTs/GQD) mixture for further use. A total of 8 μL of MWCNTs/GQD mixture was dropped onto the GCE surface and dried at room temperature for 30 min to ensure the mixture was completely dry, which was named MWCNTs/GQDs/GCE.

### 2.3. Preparation of Imprinted Electrochemical Sensor

The electropolymerization of the SMZ onto the GCE was carried out using the cyclic voltammetry technique. The above-modified MWCNTs/GQDs/GCE was immersed in acetate buffer solution (pH 5.2) containing 2 mM of SMZ and 8 mM of OPD. Then, the electrochemical polymerization was performed using cyclic voltammetry technique from 0 V to 1.5 V, with 10 cycles at a scan rate of 50 mV/s. The template molecules were removed by immersing the modified electrode in methanol/acetic acid (9/1, *v*/*v*) for 5 min, subsequently immersed in 0.5 M of H_2_SO_4_/methanol (4/1, *v*/*v*) solution and eluted with the CV method from −0.2 V to 1.4 V with 50 cycles at a scan rate of 100 mV/s. Then, the imprinted electrochemical sensor (MIPs/MWCNTs/GQDs/GCE) was prepared. Meanwhile, the nonimprinted electrochemical sensor (NIPs/MWCNTs/GQDs/GCE) was prepared with the same method as the imprinted electrochemical sensor but without SMZ in the whole process.

### 2.4. Electrochemical Measurement

All electrochemical measurements were performed with a three-electrode system in a0.1 M of KCl solution containing 5 mM of [Fe(CN)_6_]^3−/4−^. The modified electrodes were used as working electrodes, while a platinum wire served as the counter electrode and an Ag/AgCl electrode (with 3 M of KCl) was used as the reference electrode. The electrochemical properties of the modified electrode were characterized by CV. The CV measurements were conducted over a potential range of −0.2 V to 0.8 V (vs. Ag/AgCl) at a scan rate of 50 mV/s.

### 2.5. Real Sample Preparation

The proposed sensor was used to detect the sulfamethazine in the seawater to determine its applicability. The real seawater samples were collected from an aquaculture company (China). A total of 1 mL of a filtered water sample was spiked with the standard solution and then fixed to 10 mL with acetate buffer solution (ABS, pH 9). Then, the resulting mixture was used to analyze using the developed MIPs/MWCNTs/GQDs/GCE sensor through square-wave voltammetry (SWV) with a potential range of 0.4 V to 1.3 V.

## 3. Results and Discussion

### 3.1. The Preparation and Characteristics of the Molecularly Imprinted Electrochemical Sensor

**Scheme 1:**
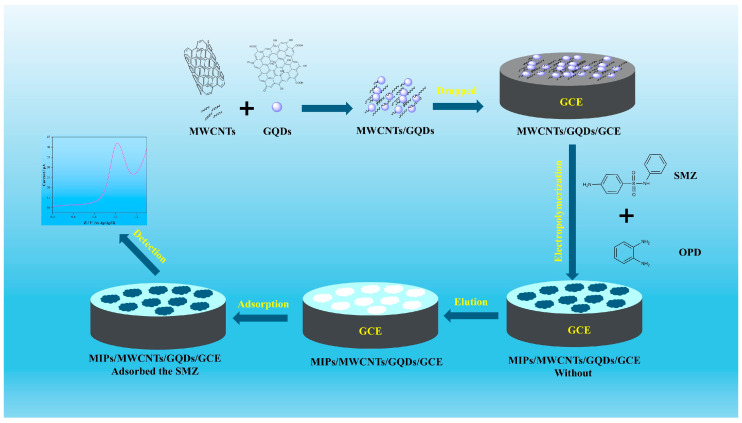
Schematic of preparation of MWCNTs/MIPs and analysis of electrochemical sensors.

The molecularly imprinted membrane was modified onto the surface of the GCE by electropolymerization, using MWCNTs/GQDs as the conductive support, SMZ as the molecular template, and OPD as the functional monomer. After eluting SMZ from the polymer, cavities of the same size, complementary shapes, and recognition sites corresponding to the SMZ molecules were present in the modified layer. When the modified electrode came into contact with SMZ again, it selectively recognized and adsorbed SMZ (Scheme 1). However, NIPs/MWCNTs/GQDs did not possess these characteristics because no template was involved in the synthesis process.

In addition, the morphology of the synthesized polymers was observed by scanning electron microscopy (SEM), and it was found that the electrodes modified with MIPs had a sparser and more porous structure compared to those modified with NIPs (Figure S2). Therefore, it has been demonstrated that MIPs/MWCNTs/GQDs/GCE, with its porous structure, provides a more effective contact area for the active material to participate in the reaction than NIPs/MWCNTs/GQDs/GCE, directly leading to its enhanced electrochemical properties.

### 3.2. Electrochemical Behavior of the Modified Electrode

**Figure 1:**
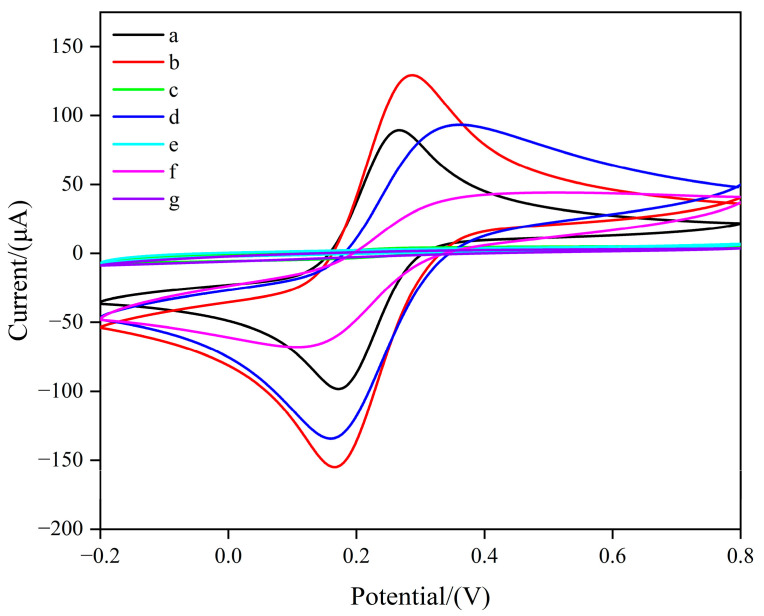
CV of different modified electrodes in the solution of 0.1 M KCl containing 0.5 mM [Fe(CN)_6_]^3−/4−^ as the electroactive species. (a) bare GCE; (b) MWCNTs/GQDs/GCE; (c) MIP/MWCNTs/GQDs/GCE before elution; (d) MIP/MWCNTs/GQDs/GCE; (e) NIP/MWCNTs/GQDs/GCE; (f) MIP/MWCNTs/GQDs/GCE after incubation in the SMZ solution; (g) NIP/MWCNTs/GQDs/GCE after incubation in the SMZ solution.

To investigate the electrochemical behavior of the modified electrodes, cyclic voltammetry (CV) was performed in a 5 mM [Fe(CN)_6_]^3−/4−^ solution containing 0.1 M KCl, over a potential range of −0.2 V to 0.8 V at a scan rate of 50 mV/s. As shown in Figure 1, compared with the GCE, the peak current increased when MWCNTs/GQDs were modified on the electrode surface. This indicates that the excellent electrical conductivity of carbon nanomaterials effectively reduced the activation energy of the electrochemical reaction and promoted the redox reaction of [Fe(CN)_6_]^3−/4−^ on the MWCNTs/GQDs/GCE surface.

When the imprinted film was electropolymerized onto the MWCNTs/GQDs/GCE surface, no redox peak current appeared in the CV scanning curve of the MIPs/MWCNTs/GQDs/GCE. This suggests that the dense imprinted film was successfully polymerized on the electrode surface, hindering the redox reaction of [Fe(CN)_6_]^3−/4−^ on the modified electrode. After elution of the MIPs/MWCNTs/GQDs/GCE, the redox peak current increased significantly, indicating that the template molecules had been successfully removed. This created specific molecularly imprinted cavities complementary to the SMZ structure, allowing the [Fe(CN)_6_]^3−/4−^ probes to diffuse into the film for redox reactions.

After immersing the MIPs/MWCNTs/GQDs/GCE in an SMZ solution, the CV peak current decreased significantly. This was mainly due to the adsorption of SMZ by the imprinted polymer film on the electrode surface, which further filled the cavities. This obstructed the effective contact between the [Fe(CN)_6_]^3−/4−^ probe and the electrode, thereby affecting electron transfer.

The electrochemical impedance spectra (EIS) of the different modified electrodes were further investigated, where the high-frequency semicircle diameters represent the charge transfer resistance (R_ct_). When the GCE surface was drop-coated with MWCNTs/GQDs, the resistance decreased significantly (R_ct_ 130.6 Ω), indicating that the modification of composite carbon nanomaterials enhanced the electrical conductivity of GCE (Figure S3). When MIPs were electropolymerized onto the surface of MWCNTs/GQDs/GCE, the diameter of their high-frequency semicircle became significantly large. The spatial site-barrier effect and insulating properties of MIPs impede electron transfer, thereby contributing to the large Rct. The diameter of the semicircle of MIPs/MWCNTs/GQDs/GCE after the elution of SMZ decreased because the presence of pores on the MIP provided a reliable pathway for electron transfer (R_ct_ 161.8 Ω), which was also consistent with the information obtained from CV.

In addition, in order to understand the effective specific surface area of the MIPs/MWCNTs/GQDs/GCE. The CV measurement was performed in 0.1 M KCl solution (containing 5.0 mM [Fe(CN)_6_]^3−/4−^). According to the Randles–Sevcik equation [57], the effective surface area of MIPs/MWCNTs/GQDs/GCE was 0.0916 cm^2^.

$$I_{p}=2.69\times 10^{5}D^{1/2}n^{3/2}AV^{1/2}C$$

where I_p_ is the response current of the modified electrode (A) which was 1.385 × 10^−4^ A with the CV test (voltage range from −0.2 V to 0.8 V, scan rate: 0.05 V/s), D is the diffusion coefficient (7.6 × 10^−5^ cm^2^/s), n is the number of transferred electrons, A is the surface area of the modified electrode (cm^2^), V is the scan rate (V/s), and C is the concentration of electroactive species (5 × 10^−6^ mol/L).

### 3.3. Electrochemical Action of the MIP/MWCNTs/GQDs/GCE During SMZ Oxidation

**Figure 2:**
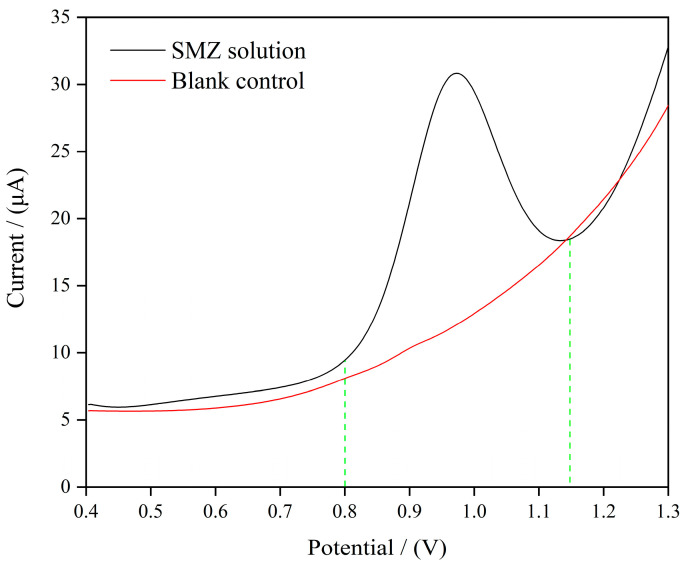
SWV recorded in ABS (pH 9.0) contained 100 µM of SMZ with potential varying from 0.4 V to 1.3 V and the blank control solution (The green line represents the voltage range of SMZ electrocatalytic oxidation).

The electrochemical oxidation of SMZ on the MIP/MWCNTs/GQDs/GCE was performed in ABS at pH 9 using square-wave voltammetry (SWV). As depicted in Figure 2, the addition of 100 μM of SMZ to the electrochemical cell elicited a distinct oxidation process. The anodic peak was observed in the potential range of 0.8 to 1.15 V versus the Ag/AgCl silver/silver reference electrode. This phenomenon can be ascribed to the electrochemical reaction involving the primary amino group of SMZ, in which one proton and one electron participate. This outcome facilitated the direct and precise determination of SMZ in the sample.

### 3.4. Optimization of the Modified Sensors and Determination

#### 3.4.1. The Amount of Composite Carbon Nanomaterials

Firstly, the optimal drop-coating volume of composite carbon nanomaterials used for modifying the GCE was investigated. Different volumes of MWCNTs/GQDs chitosan suspensions were applied to the surface of the GCE, and after vacuum drying at 60 °C, the peak-current response of the modified electrode in [Fe(CN)_6_]^3−/4−^ was measured using cyclic voltammetry (CV).

**Figure 3:**
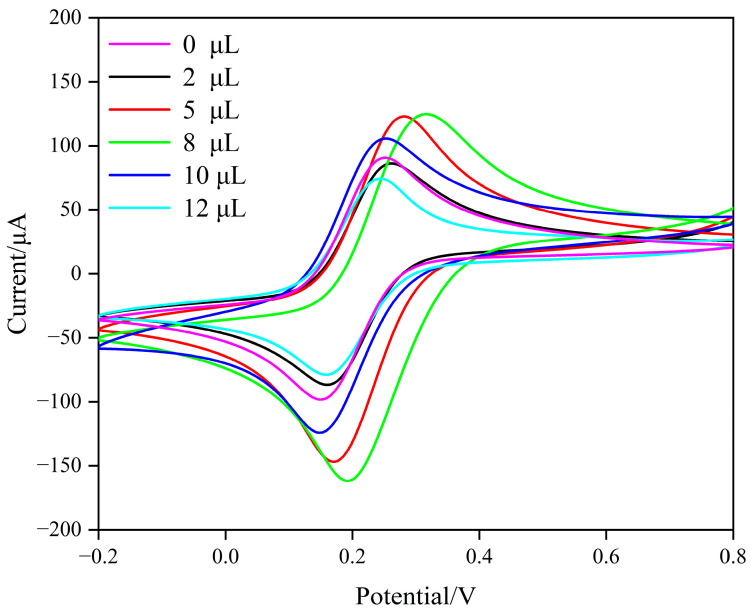
CV of composite carbon nanomaterials with different amounts of dropwise coating in the solution of 0.1 M KCl containing 0.5 mM [Fe(CN)_6_]^3−/4−^ as the electroactive species.

When 8 μL of composite carbon nanomaterials was applied to the GCE, the modified electrode exhibited better electrochemical performance (Figure 3). When the volume was too small, the composite carbon nanomaterials were insufficient to cover the entire electrode surface, reducing surface utilization and limiting the redox probe’s interaction, which resulted in a minimal improvement in current response. Excessive coating increased the thickness of the electrode modification layer, reducing the effective contact area between the probe and the electrode, which led to a decrease in current response [58]. Therefore, 8 μL of composite carbon nanosuspension was selected as the optimal drop-coating volume for the preparation of imprinted electrochemical sensors.

#### 3.4.2. The pH of the Electrolyte

**Figure 4:**
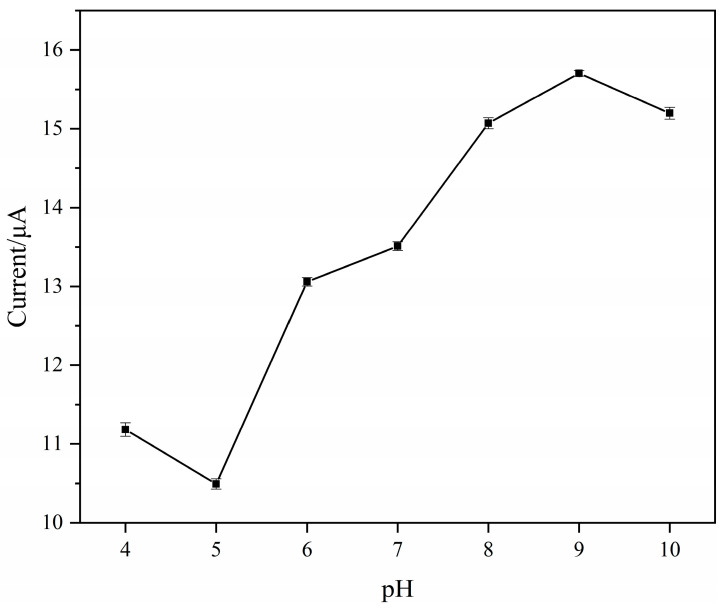
The current response of MIPs/MWCNTs/GQDs/GCE in ABS solutions with different pH values (including 80 μM SMZ as the electroactive species).

The pH of the electrolyte not only affects the current response of the electrode but also affects the stability of the molecularly imprinted membrane modified on the surface during electrochemical detection. Therefore, it is necessary to choose a suitable electrolyte pH to protect the electrode modification layer from being damaged, and to have a stable current response during the detection. The electrochemical behavior of the modified electrode was investigated using SWV, and its current response in ABS electrolyte (containing 80 μM of SMZ) at a different pH was evaluated (Figure 4), in which SMZ was the redox probe. The sensor exhibited a high current response to SMZ when the solution pH was 9, which was attributed to the present form of SMZ in solution binding of the anionic form with the pKa of 7.65, and might enable combination with the binding sites in the MIPs/MWCNTs/GQDs/GCE sensor through hydrogen bonds. However, it was found that when the solution was acidic, containing acetic acid, the surface-modified imprinted film was gradually detached after the electrode was subjected to multiple SWV measurements, which affected the detection. When the solution was more alkaline, the adsorption effect of the imprinted polymeric film on the surface of the electrode on SMZ was affected, resulting in a lower current response. Therefore, an ABS buffer solution of pH 9 was selected as the supporting electrolyte for subsequent work.

#### 3.4.3. Optimization of Elution Method

The efficient elution of adsorbed SMZ from the modified electrode is a pivotal step in the process of electrode regeneration and reuse. Currently, the primary methods for template elution from modified electrodes include electrochemical elution and organic solvent elution. A range of solvents, including methanol/acetic acid, ethanol/acetic acid, methanol/sodium hydroxide, and ethanol/sodium hydroxide, were evaluated by immersing the MIPs/MWCNTs/GQDs/GCE that had been saturated with SMZ (Supplementary Materials S1). It was found that soaking in methanol/acetic acid (*v*/*v*, 9/1) resulted in noticeable current recovery (Figure S4); in addition, the SMZ adsorbed on its surface was eluted to a stable state when the modified electrode was immersed for five minutes (Figure S5). However, the response current remained relatively low, indicating that simple chemical soaking is insufficient to completely elute SMZ from the surface of the imprinted modified electrode.

Therefore, a combined approach of organic solvent elution and electrochemical elution was considered. The MIPs/MWCNTs/GQDs/GCE adsorbed with SMZ was first soaked in methanol/acetic acid (9/1, *v*/*v*) for 5 min, followed by cyclic voltammetry scanning in 0.5 M of H_2_SO_4_/methanol (4/1, *v*/*v*) with a voltage range of −0.4 V to 1.4 V, a scan rate of 0.1 V/s, and varying the number of scan cycles. After elution, the modified electrode was subjected to CV testing with [Fe(CN)_6_]^3−/4−^ as the redox probe.

**Figure 5:**
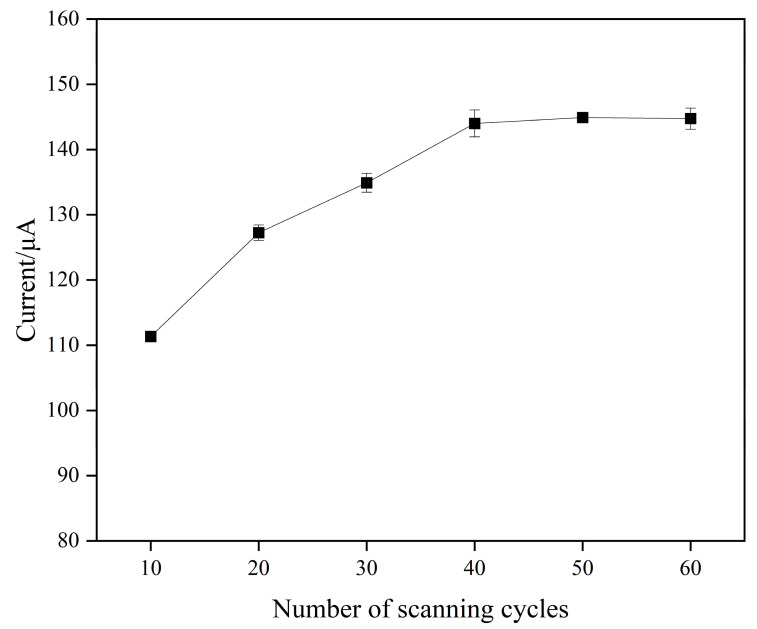
The current response of modified electrodes with different number of scanning cycles for elution in the solution of 0.1 M KCl containing 0.5 mM [Fe(CN)_6_]^3−/4−^ as the electroactive species.

The results showed that the response current of the MIPs/MWCNTs/GQDs/GCE gradually increased with the number of scanned cycles and stabilized when the number of cycles reached 40 (Figure 5). To ensure complete elution of SMZ from the modified electrode surface, 50 scan cycles were chosen as the optimal elution condition.

#### 3.4.4. Standard Solution Analysis

**Figure 6:**
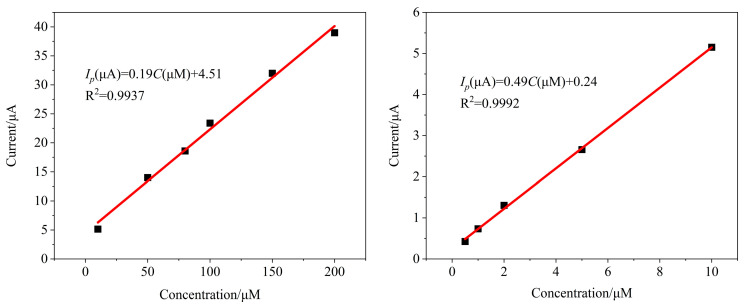
Linear curves of MIPs/MWCNTs/GQDs/GCE in different concentrations of SMZ standard solutions (0.5–200 μM of SMZ as the electroactive species).

Under optimal detection conditions, the SWV responses of different concentrations of SMZ were recorded using the MIPs/MWCNTs/GQDs/GCE sensor. As shown in Figure 6, the current response (Ip) of SMZ increased with increasing concentration, exhibiting linear correlations in the ranges of 0.5–10 μM and 10–200 μM. The linear equations were Ip = 0.49C + 0.24 and Ip = 0.18C + 4.51, respectively. The calculated LOD was 0.068 μM (S/N = 3), and the RSD was less than 5%, indicating that the method established using the MIPs/MWCNTs/GQDs/GCE sensor demonstrates excellent performance for SMZ analysis. In addition, we compared the proposed method with other methods for detecting SMZ (Table S2) [12,59,60,61,62]. The proposed method was found to have a satisfactory LOD, which had a high potential for application.

#### 3.4.5. Selectivity of the Sensor

**Figure 7:**
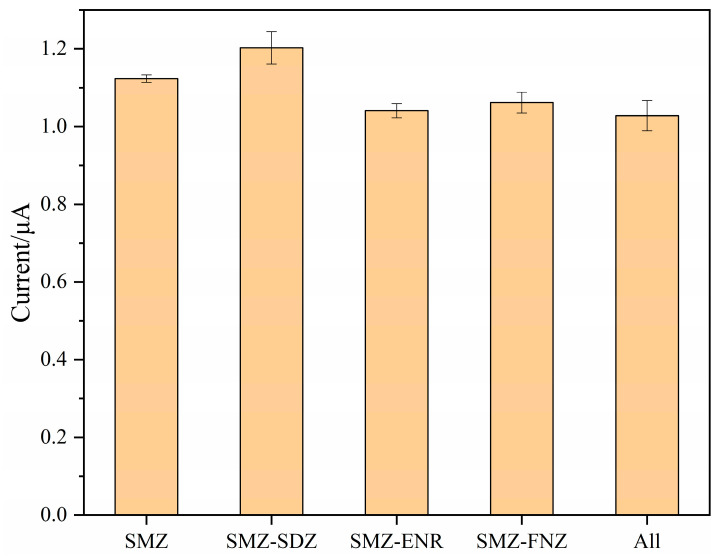
The response current to SMZ and other interfering compounds (SMZ was the electroactive species).

To investigate the anti-interference ability and specific recognition of SMZ by the MIPs/MWCNTs/GQDs/GCE sensor, a selectivity study was conducted. Sulfadiazine (SDZ), a structural analog of SMZ, was chosen as the competing compound, while enrofloxacin (ENR) and furazolidone (FNZ), which are not structurally similar to SMZ, were selected as interference compounds. A mixed standard solution containing 50 μM of both SMZ and the competing substances was prepared using an ABS buffer solution (pH 9) as the solvent. SWV measurements were then performed for each solution, and the results are shown in Figure 7 and Figure S6.

The response current of the MIPs/MWCNTs/GQDs/GCE sensor in the single SMZ solution was compared with that in the mixed solution of competing substances. No significant difference in the current response was observed, and the addition of competing substances did not affect the electrode’s response to SMZ. This indicates that the MIPs/MWCNTs/GQDs/GCE sensor possesses excellent anti-interference ability.

#### 3.4.6. Reusability and Stability

**Figure 8:**
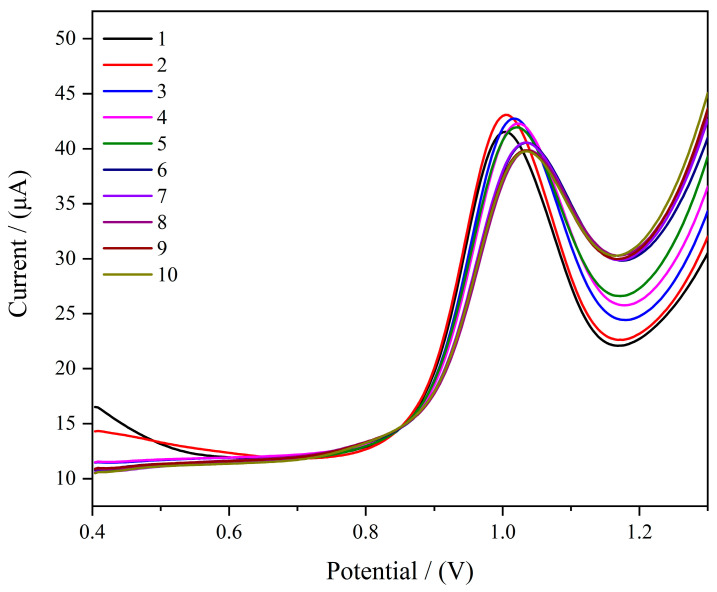
SWV curves of consecutive detections of SMZ using MIPs/MWCNTs/GQDs/GCE. (SMZ was the electroactive species).

Reusability is a key advantage of molecularly imprinted electrochemical sensors. To further explore the reproducibility and stability of the MIPs/MWCNTs/GQDs/GCE electrochemical sensor, the used modified electrode was regenerated and reused by eluting it with CV in 20 mL of 0.5 M of H_2_SO_4_/methanol (4/1, *v*/*v*) solution for 20 cycles. The voltage range was 0.4 V to 1.3 V, with a scan rate of 0.1 V/s. The regenerated modified electrode was then used to perform SWV analysis on a 100 μM SMZ standard solution to evaluate its reusability. The results are shown in Figure 8. The prepared imprinted electrochemical sensor maintained a high response current even after 10 cycles of reuse, with a relative standard deviation of 1.86%, indicating that the modified electrode exhibits high reusability.

Meanwhile, the stability of MIPs/MWCNTs/GQDs/GCE was investigated by using three modified electrodes to detect SMZ standard solution (100 μM) every 24 h for five consecutive days (Figure S7). The RSD of response-current fluctuation was 3.93%, respectively, indicating that the molecularly imprinted electrochemical sensor had satisfactory stability.

#### 3.4.7. Determination of Real Samples

After filtering the aquaculture seawater through a 0.22 μM membrane, 50 µL of SMZ standard solution (10 mM) was added to 1 mL of the water sample, which was then fixed to 10 mL using the ABS buffer solution (pH 9). The spiked samples were detected using SWV, and the SMZ content in the detection solution was calculated using the linear regression equation. The results, shown in Table 1 and Figure S8, indicate that no SMZ residues were detected in any of the six water samples. The spiked recovery rates ranged from 95.4% to 104.8%, with a relative standard deviation (RSD) of less than 4.14%. It was demonstrated that the developed imprinted electrochemical sensor performed well in detecting SMZ in actual aquaculture seawater samples. Finally, SMZ was not detected in the collected water samples, indicating that there was no SMZ contamination in the aquaculture water environment, or that the SMZ contained was below the detection limit, and the level of SMZ contamination is too low to be of concern.

## 4. Conclusions

In summary, this study successfully developed a novel molecularly imprinted electrochemical sensor based on a composite of multi-walled carbon nanotubes (MWCNTs) and graphene quantum dots (GQDs) for the detection of sulfamethazine (SMZ) in aquaculture seawater. The sensor demonstrated remarkable performance, showcasing high sensitivity, selectivity, and reproducibility.

The integration of MWCNTs and GQDs onto the glassy carbon electrode (GCE) significantly enhanced the electrical conductivity and effective surface area of the sensor, leading to an improved signal-to-noise ratio and increased sensitivity for SMZ detection. The electropolymerization process, utilizing cyclic voltammetry, effectively formed a dense imprinted film on the electrode surface that specifically recognized and bound to SMZ molecules.

The optimized conditions, including the amount of composite carbon nanomaterials, the pH of the electrolyte, and the elution method, were crucial for achieving maximum sensor performance. Under these conditions, the sensor exhibited linear responses to SMZ concentrations in the ranges of 0.5–10 μM and 10–200 μM, with a low limit of detection (LOD) of 0.068 μM. Furthermore, the sensor demonstrated excellent selectivity, with minimal interference from both structurally similar and dissimilar compounds.

The reusability of the sensor was confirmed through consecutive detection cycles, maintaining a high response current and low relative standard deviation. In practical applications, the sensor was successfully applied to detect SMZ residues in aquaculture seawater samples, with spiked recovery rates ranging from 95.4% to 104.8% and relative standard deviations below 4.14%.

Overall, the developed MIPs/MWCNTs/GQDs/GCE sensor represents a promising tool for the rapid, sensitive, and selective detection of SMZ in aquaculture environments, contributing to the safeguarding of public health and the protection of aquaculture. This study provides valuable insights into the potential of molecularly imprinted electrochemical sensors for antibiotic residue monitoring in aquaculture systems.

## Figures and Tables

**Table 1:** Spiked recovery of aquaculture seawater samples for the detection of SMZ with MIPs/MWCNTs/GQDs/GCE sensor (n = 3).

Aquaculture Seawater Samples		Spiked Concentration (μM)	Detection Concentration (μM)	Rate of Recovery (%)	RSD (%)
*Epinephelus fasciatus*	Natural seawater	0	Nd ^a^	/	/
		50	47.9	95.9	0.61
	Culture water	0	Nd	/	/
		50	51.9	103.9	2.62
	Tailwater	0	Nd	/	/
		50	50.6	101.2	2.02
*Epinephelus* sp.	Natural seawater	0	Nd	/	/
		50	52.4	104.8	3.19
	Culture water	0	Nd	/	/
		50	47.7	95.4	4.47
	Tailwater	0	Nd	/	/
		50	49.7	99.4	3.11

Nd: not detected.

## Data Availability

The raw data supporting the conclusions of this article will be made available by the authors on request.

## Supplementary Materials

The following supporting information can be downloaded at: https://www.mdpi.com/article/10.3390/bios15030184/s1, Figure S1: XRD pattern of MWCNTs and GQDs; Figure S2: The SEM of electrode modified films of MIPs (A) and NIPs (B); Figure S3: EIS of the different modified electrodes in the solution of 0.1 M KCl containing 0.5 mM. [Fe(CN)_6_]^3−/4−^ as the electroactive species (a, GCE; b, MWCNTs/GQDs/GCE; c, MIPs/MWCNTs/GQDs/GCE before removing the SMZ; d, MIP/MWCNTs/GQDs/GCE after removing the SMZ); Figure S4: Current response of modified electrodes in different types of the eluent in the solution of 0.1 M KCl containing 0.5 mM. [Fe(CN)_6_]^3−/4−^ as the electroactive species; Figure S5: Different times of the elution with immersion in methanol/acetic acid in the solution of 0.1 M KCl containing 0.5 mM. [Fe(CN)_6_]^3−/4−^ as the electroactive species; Figure S6: SWV curves for the interfering compounds with SMZ; Figure S7: Stability of the MIPs/MWCNTs/GQDs/GCE sensors (a) and the SWV curves (b); Figure S8: SWV curves of real samples. (The uppercase A-F represented the sample with a spiked concentration of 0 μM (i.e., the actual sample), while the lowercase a-f represented the sample with a spiked concentration of 50 μM; A/a-C/c were natural seawater, culture water, tailwater of *Epinephelus fasciatus*; D/d-F/f were natural seawater, culture water, tailwater of *Epinephelus* sp.); Table S1: Comparison of MIP Sensors for Sulfamethazine (SMZ); Table S2: Comparison of the proposed method with other methods.
