# Supplementary material for: Molecularly Imprinted Electrochemical Sensor Based on MWCNTs/GQDs for the Detection of Sulfamethazine in Aquaculture Seawater
Source: Biosensors (Basel). 2025 Mar 13;15(3):184. doi: 10.3390/bios15030184 (PMC11940161; doi:10.3390/bios15030184)
Supplement: Supplementary file 1 [file biosensors-15-00184-s001.zip › biosensors-3409512-supplementary.pdf]

Molecularly Imprinted Electrochemical Sensor Based on MWCNTs/GQDs for the Detection of Sulfamethazine in Aquaculture Seawater

Jianlei Chen [1,2], Tianruo Zhang [1,2,3], Yong Xu [1,2], Hao Li [1,2], Hongwu Cui [1,2], Xinguo Zhao [1,2], Yun Zhou [1,2,3], Keming Qu [1,2] and Zhengguo Cui [1,2,*]

1 State Key Laboratory of Mariculture Biobreeding and Sustainable Goods, Yellow Sea Fisheries Research Institute, Chinese Academy of Fishery Sciences, Qingdao 266071, China; chenjl@ysfri.ac.cn (J.C.)
2 Laboratory for Marine Fisheries Science and Food Production Processes, Qingdao Marine Science and Technology Center, Qingdao 266237, China
3 College of Fisheries and Life Science, Dalian Ocean University, Dalian 116023, China
* Correspondence: cuizg@ysfri.ac.cn; Tel.: +86-532-85836341

S1. Eluent type and elution time

Different types of organic eluents were investigated in this study, such as methanol/acetic acid (V/V, 9/1 and 8/2), ethanol/acetic acid (V/V, 9/1 and 8/2), methanol/0.5mol/L of NaOH (V/V, 9/1), and ethanol/0.5mol/L of NaOH (V/V, 9/1) mixtures at different volume ratios. The adsorbed SMZ sensors were immersed in the corresponding solutions for 10 min. Afterwards, the CV test were measured in 0.1mol/L of KCl containing 0.5 mmol/L $[Fe(CN)6]^{3-/4-}$. The result was shown in Figure S4. The effect of immersion in methanol/acetic acid for different times (0.5 min-10 min) on the elution of modified electrodes was further investigated. The result was shown in Figure S5.

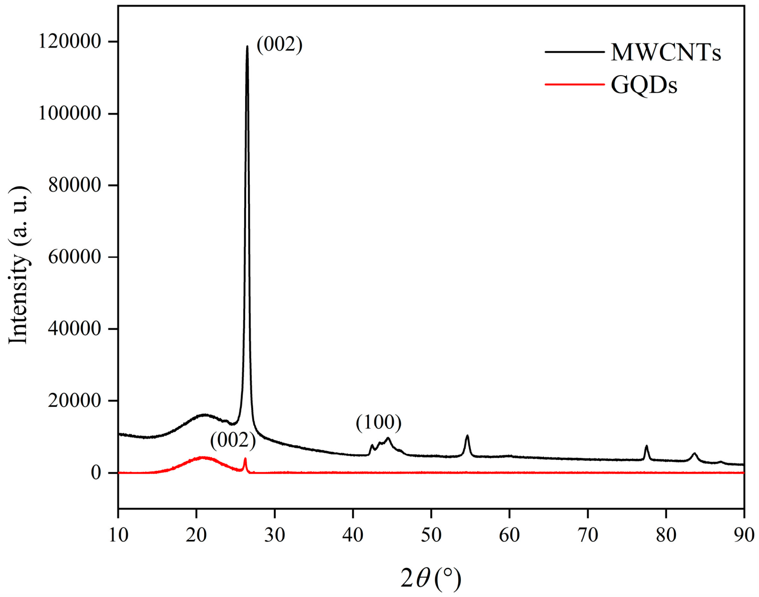

Figure S1 XRD pattern of MWCNTs and GQDs

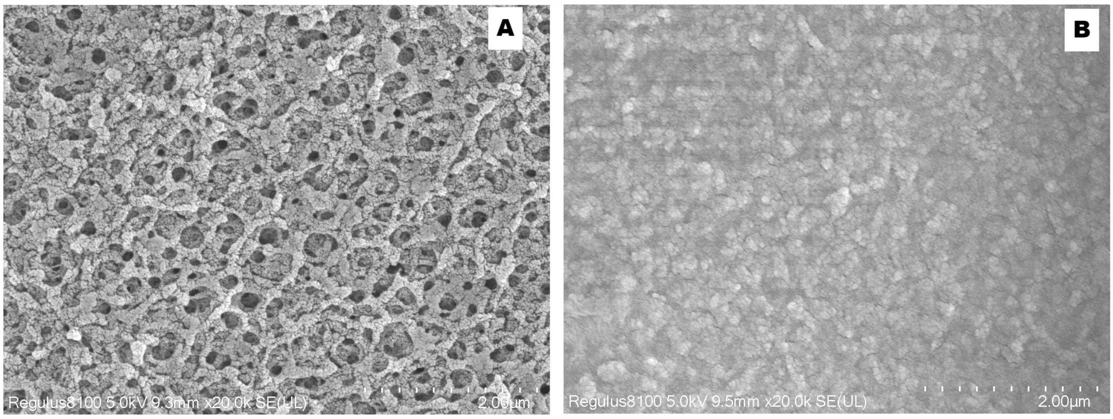

Figure S2. The SEM of electrode modified films of MIPs (A) and NIPs (B)

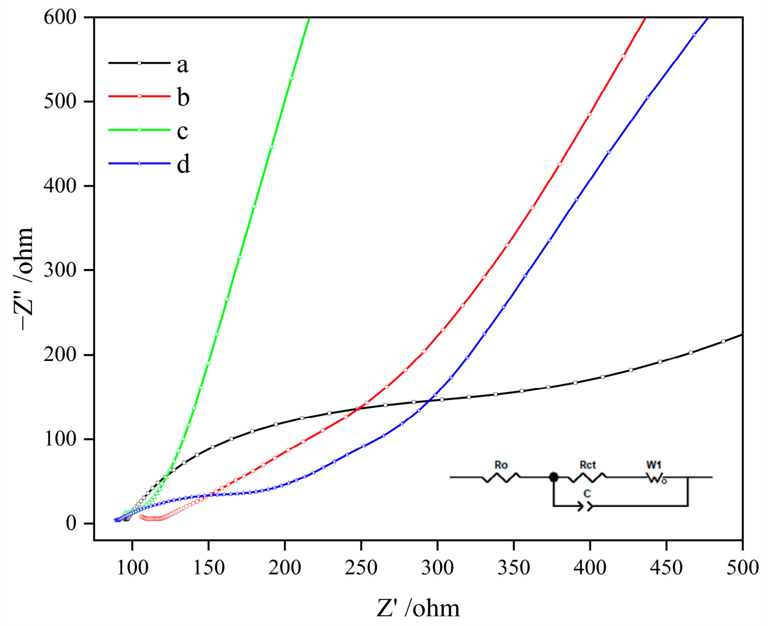

Figure S3 EIS of the different modified electrodes in the solution of 0.1 M KCl containing 0.5 mM. $[Fe(CN)_6]^{3-/4-}$ as the electroactive species (a, GCE; b, MWCNTs/GQDs/GCE; c, MIPs/ MWCNTs/GQDs/GCE before removing the SMZ; d, MIP/MWCNTs/GQDs/GCE after removing the SMZ)

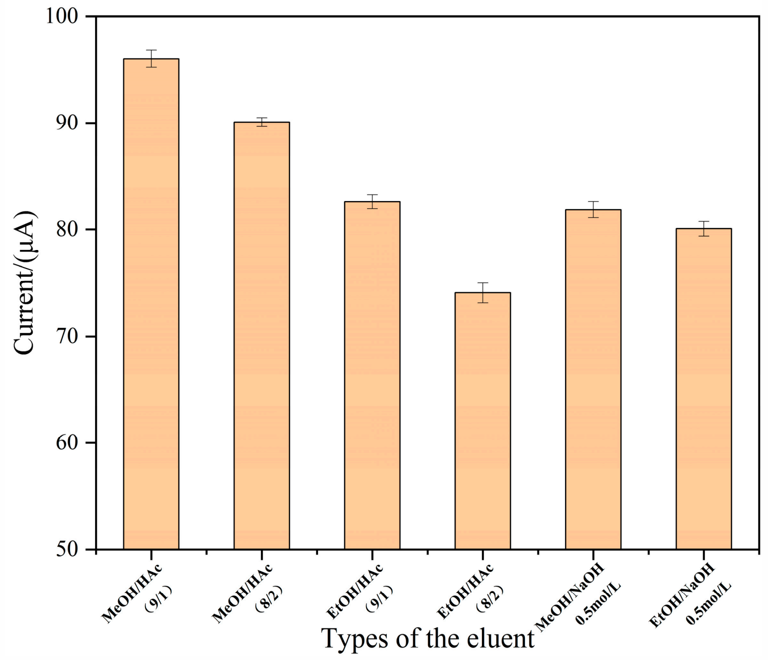

Figure S4. Current response of modified electrodes in different types of the eluent in the solution of 0.1 M KCl containing 0.5 mM. $[Fe(CN)_6]^{3-/4-}$ as the electroactive species

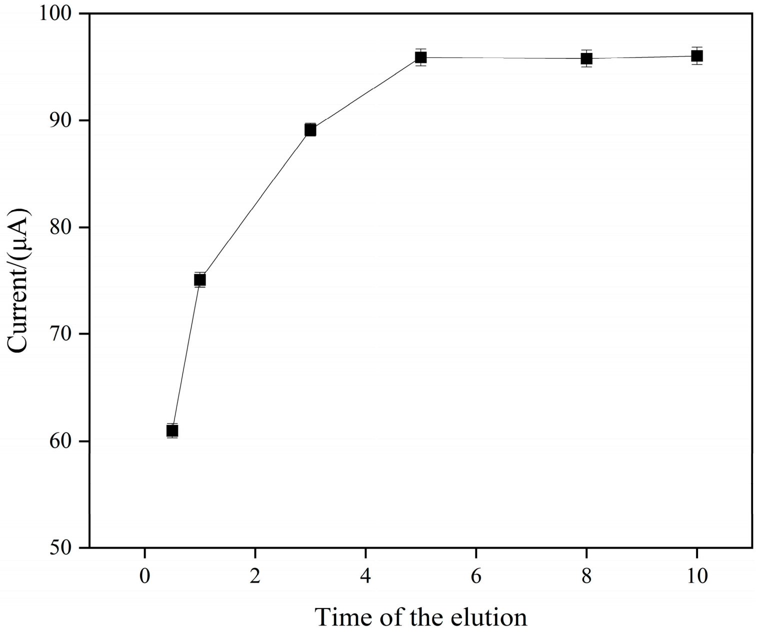

Figure S5. Different times of the elution with immersion in methanol/acetic acid in the solution of 0.1 M KCl containing 0.5 mM. $[Fe(CN)_6]^{3-/4-}$ as the electroactive species

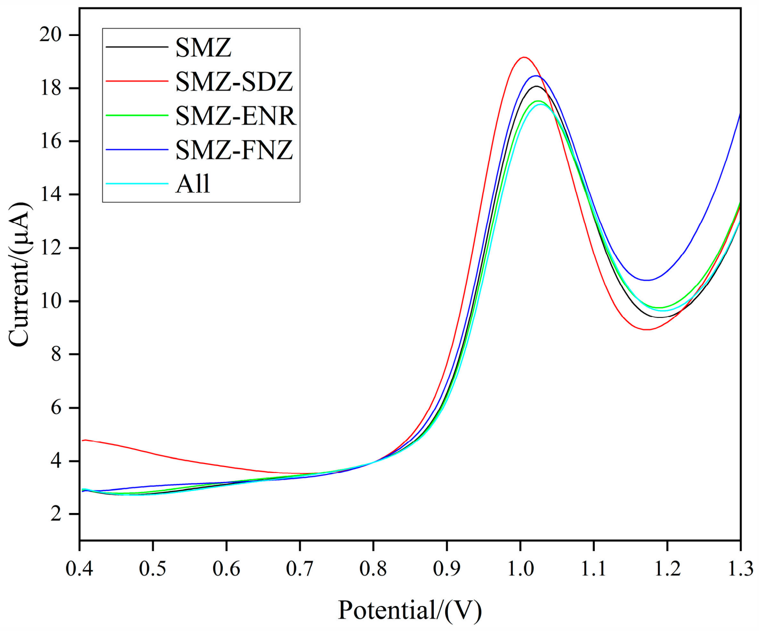

Figure S6. SWV curves for the interfering compounds with SMZ

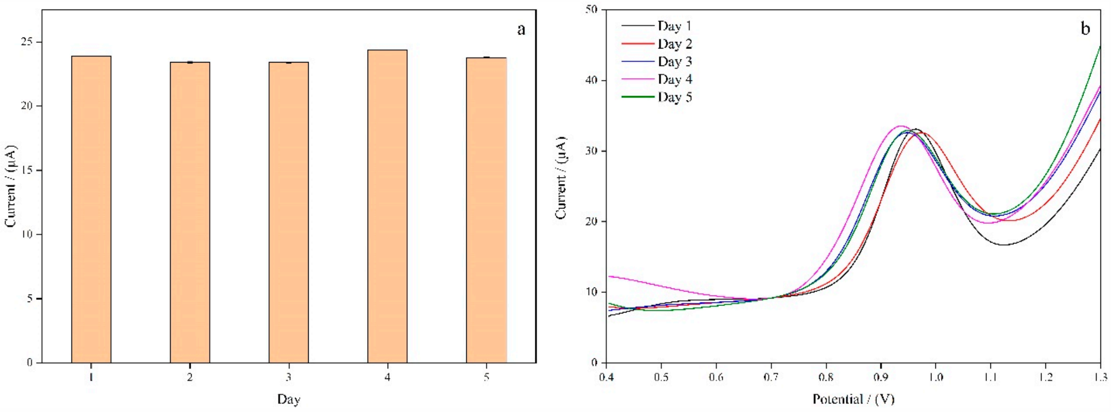

Figure S7 Stability of the MIPs/MWCNTs/GQDs/GCE sensors (a) and the SWV curves (b)

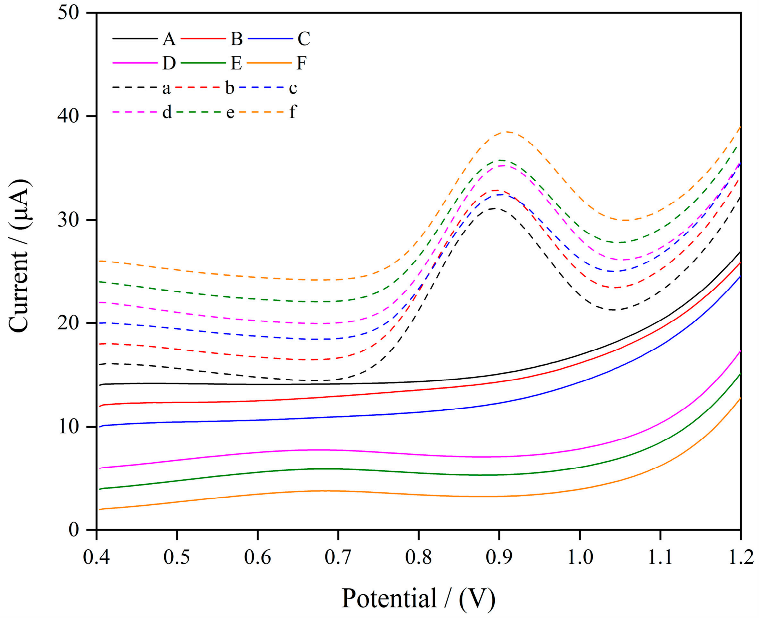

Figure S8. SWV curves of real samples. (The uppercase A-F represented the sample with a spiked concentration of 0 μM (i.e., the actual sample), while the lowercase a-f represented the sample with a spiked concentration of 50 μM; A/a-C/c were natural seawater, culture water, tailwater of *Epinephelus fasciatus*; D/d-F/f were natural seawater, culture water, tailwater of *Epinephelus sp.*)

Table S1 Comparison of MIP Sensors for Sulfamethazine (SMZ)

NO.	Materials	Sensors	LOD	Detection range	Real Samples	Response Time/ Practical Notes	References
1	ZnO@Fe_3O_4 MIP	Fluorescence spectra	19 μg/L	0-10μM	Pork meat	fluorescence quenching immediate, but sample required extraction	Xu et al., 2012
2	photonic crystal MIP	Fiber optic spectrometer	1.16μg/L	0.1μg/L-10mg/L	Milk & chicken	visible color change within 3 min	He et al., 2025
3	Thermosensitive core–shell MIP on SiO_2	Adsorption study; not a direct sensor	/	/	/	/	Huang et al., 2018
4	Inverse opal photonic MIP hydrogel	Fiber optic spectrometer	/	3.6μM - 18μM	Egg white	~5 min	Zhang et al., 2018
5	AgNP-decorated MIP	Surface-enhanced Raman scattering	0.081 nM	0.1 nM – 1μM	Lake water	Fast detection: (SERS spectrum in seconds),<br>requires Raman instrument	Jiang et al., 2023
6	MIP@ZnO	Fluorescent sensor	0.62 μM	0–40 μM	Milk & tap water	fast response,<br>direct fluorescence readout	Zheng et al., 2020
7	MIP/CNT/ MoS2-CoNi/GCE	Electrochemical sensor	0.033μM	0.1-800 μM	Meat	Fast analysis: DPV measurement in seconds	Niu et al., 2025
8	PCMIP@CDs	Fluorescence	/	0.01-0.26nM	Water & Fish	~3.3 min	Zhang et al., 2023
9	MIP/v-COF@SWCNTs-COOH/SPCE	Electrochemical sensor	0.21 nM		Milk & Fish	Fast analysis: DPV scan in <1 min	Bai et al., 2025
10	PCMIP sensor array	Reflectance	0.28 nM	10 nM-100 μM	Fish meat	Rapid	Lin et al., 2020
11	ZnS:Mn QD@MIP	Fluorescent	0.5 μM	0-80 μM	Tap water	Rapid	Hu et al., 2018
12	MIPs@QDs@SiO2	Fluorescence emission spectra	0.78μM	10-60μM	Honey	Moderate speed: fluorescence readout in minutes	Cao et al., 2024

Notes: SMZ = sulfamethazine (also called sulfadimidine, SM2); GCE = glass carbon electrode; SPCE = screen-printed carbon electrode; COF = covalent organic framework; QDs = quantum dots; SERS = surface-enhanced Raman scattering. LOD = limit of detection (typically S/N = 3).

Table S2 Comparison of the proposed method with other methods

Methods	Materials	Linear range (μM)	Detection limit (μM)	Type of sample	Reference
Fluorescence	MIP	9-54	9	Milk	Chen et al., 2018
HPLC	MIP-silica	0.35-79.6	0.089	Milk	Su et al., 2008
DPASV	AuNP/TCBt/GCE	0.89-107.7	0.097	Spring water	Lalmalsawmi et al., 2022
CV	MWCNT-GCE	10-200, 300-3000	6.1	Serum	Lida and Masoume, 2014
DPV	GCE/rGO-AuNPs	0.5-6.5	0.1	Effluent	Silva and Cesarino, 2019
SWV	MIPs/MWCNTs/GQDs/GCE	0.5-200	0.068	Seawater	This work

References

Chen, L., Xu, Y., Sun, L., Zheng, J., Dai, J., Li, C., Yan, Y. 2018. Convenient determination of sulfamethazine in milk by novel ratiometric fluorescence with carbon and quantum dots with on-site naked-eye detection and low interferences, *Analytical Letter*, **51**, 2099–2113,

Lalmalsawmi, J., Tiwari, D., Lee, S.M., Kim, D.J., Kim, H. 2022. Efficient electrochemical sensor for trace detection of sulfamethazine in spring water: Use of novel nanocomposite material coated with Ag or Au nanoparticles. *Microchemical Journal*, **179**, 107520.

Lida, F., Masoume, Z. 2014. Electrochemical oxidation of sulfamethazine on multi-walled nanotube film coated glassy carbon electrode. *Journal of Nanostructures*, ***4***, 161–166.

Silva, M., Cesarino, I. 2019. Evaluation of a nanocomposite based on reduced graphene oxide and gold nanoparticles as an electrochemical platform for detection of sulfamethazine, *Journal of Composites Science*, **3**, 59.

Su, S., Zhang, M., Li, B., Zhang, H., Dong, X. 2008. HPLC determination of sulfamethazine in milk using surface-imprinted silica synthesized with iniferter technique, *Talanta*, **76**, 1141–1146,

Bai, S., Yang, T., Liu, P., Tan, J., Chen, S., Lei, H., Wei, X. 2025. Preparation of a V–COF@SWCNTs-COOH/SPCE supported molecularly imprinted electrochemical sensor for real-time detection of trace sulfadimidine. *Talanta*, **282**, 127046.

Cao, L., Ying, H., Zhang, B., Cao, Y., Li, S., Huang, W., Yang, W. 2024. Optimization and performance evaluation of a fluorescent sensor for residual sulfonamide antibiotics in honey samples. *Polymers for Advanced Technologies*, **35**, e6351.

He, J., Wu, M., Wang, X., Xu, R., Zhang, S., Zhao, X. 2025. Development of Molecularly Imprinted Photonic Crystals Sensor for High-Sensitivity, Rapid Detection of Sulfamethazine in Food Samples. *Polymers*, **17**, 160.

Hu, Y., Li, X., Liu, J., Wu, M., Li, M., Zang, X. 2018. One-pot synthesis of a fluorescent molecularly imprinted nanosensor for highly selective detection of sulfapyridine in water. *Analytical Methods*, **10**, 402–411.

Huang, W., Qing, Y., Wang, N., Lu, Y., Liu, T., Liu, T., Yang, W., Li S. 2018. Novel Thermosensitive Core–Shell Surface Molecularly Imprinted Polymers Based on SiO_2 for the Selective Adsorption of Sulfamethazine. *Materials*, **11**, 2067.

Jiang, G., Liu, L., Wan, Y., Li, J., Pi, F. 2023. Surface-enhanced Raman scattering based determination on sulfamethazine using molecularly imprinted polymers decorated with silver nanoparticles. *Microchimica Acta*, **190**, 169.

Lin, Z., Li, L., Fu, G., Lai, Z., Peng, A., Huang, Z. 2020. Molecularly imprinted polymer-based photonic crystal sensor array for the discrimination of sulfonamides. *Analytica Chimica Acta,* **1101**, 32–40.

Niu, Z., Shi, Y., Liu, S., Lv, Y., Wang, S. 2025. DFT-assisted design of a electrochemical sensor based on MIP/CNT/MoS2-CoNi for the detection of sulfamethazine in meat. *Journal of Food Composition and Analysis*, **140**, 107261

Xu, L., Pan, J., Dai, J., Cao, Z., Hang, H., Li, X., Yan, Y. 2012. Magnetic ZnO surface-imprinted polymers prepared by ARGET ATRP and the application for antibiotics selective recognition. *RSC Advances*, **2**, 5571-5579.

Zhang, S., Shao, K., Hong, C., Chen, S., Lin, Z., Huang, Z., Lai, Z. 2023. Fluorimetric identification of sulfonamides by carbon dots embedded photonic crystal molecularly imprinted sensor array. *Food Chemistry*, **407**, 135045

Zhang, Y., Ren, H., Yu, L. 2018. Development of molecularly imprinted photonic polymers for sensing of sulfonamides in egg white. Analytical Methods, **10**, 101

Zheng, J., Cheng, K., Wu, Y., Yu, P. 2020. Environment-friendly ZnO-based molecularly imprinting polymers fluorescence sensor for direct detection of sulfadimidine. Journal of Materials Science: Materials in Electronics, **31**, 9550-9558.
